# Preparation, characterisation, and testing of reservoir-based implantable devices loaded with tizanidine and lidocaine

**DOI:** 10.1007/s13346-025-01855-3

**Published:** 2025-04-15

**Authors:** Camila J. Picco, Mihir S. Bhalerao, Octavio E. Fandino, Elizabeth R. Magill, Qonita Kurnia Anjani, Jonathan G. Acheson, Ryan F. Donnelly, Juan Domínguez-Robles, Eneko Larrañeta

**Affiliations:** 1 School of Pharmacy, Queen’s University Belfast, Belfast, UK; 2 School of Engineering, Ulster University, Belfast, UK; 3 Department of Pharmacy and Pharmaceutical Technology, Faculty of Pharmacy, University of Seville, Seville, 41012 Spain

**Keywords:** Implantable device, Tizanidine, Lidocaine, Multiple sclerosis, Vacuum compression moulding

## Abstract

Multiple sclerosis is a chronic neuroimmunological disorder that causes progressive disability, primarily in young adults. It places a significant burden on healthcare systems due to high medication costs and long-term care needs. Implantable devices offer a promising alternative for delivering sustained drug doses in the treatment of chronic conditions. This study introduces a novel long-acting subcutaneous implant for dual-drug delivery: tizanidine (TZ) for spasticity management and lidocaine (LD) for post-insertion pain relief. Reservoir-type implants were developed with TZ in the core and LD in the shell. Two fabrication methods—direct compression and vacuum compression moulding (VCM)—were evaluated for TZ-loaded pellets (3 mm diameter, ~ 10 mm length) using TZ base and TZ hydrochloride. Pellets were encapsulated inside a biodegradable polycaprolactone (PCL) tubular membrane to control drug release. Direct compression pellets, made with poly(vinyl pyrrolidone) and hydroxypropyl-β-cyclodextrin, disintegrated quickly, releasing TZ over 20 days. VCM pellets, formulated with PCL or PCL/poly(ethylene glycol) (PEG), offered prolonged release: up to 200 days for TZ base and 80 days for TZ hydrochloride. Adding PEG accelerated TZ release, reducing duration to 20 days (TZ base) and 125 days (TZ hydrochloride). LD was incorporated into the PCL membrane, providing up to three days of sustained release. Physicochemical analysis confirmed formulation homogeneity and no covalent interactions. These findings highlight the potential of this implant system for MS-related spasticity management, supporting further research into long-acting implants to improve treatment adherence and patient outcomes.

**Graphical Abstract:**

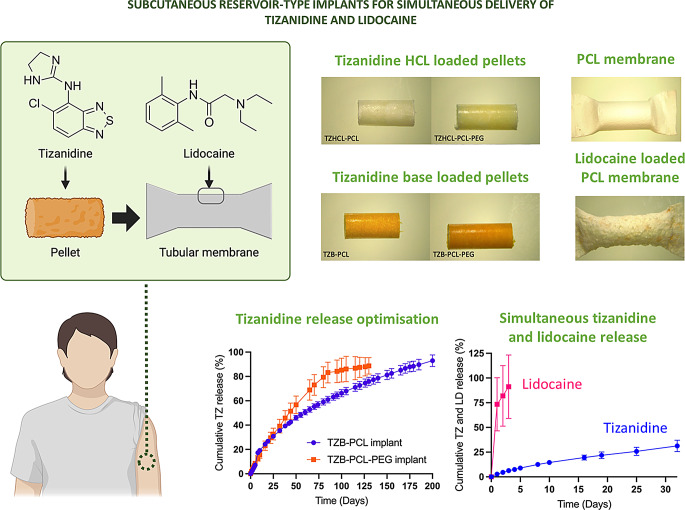

## Introduction

Multiple sclerosis (MS) is the most prominent neuroimmunology disorder [1, 2]. The disease is defined by lengthy survival and progressive disability over time, occurring in young adults most of the cases [1]. Multiple sclerosis is placing a growing burden on health-care resources because of the high cost of medication, the necessity of ongoing care, and the requirement for rehabilitation in the latter stages of the disease [2]. Spasticity is the main symptom of MS, characterised by increased muscular tone, resistance to movement, and reduced reflex function, leading to symptoms such as muscle stiffness, involuntary contractions, fibrosis, and atrophy. Spasticity causes pain and reduces movement and mobility, making it difficult to perform daily activities like walking, sitting, and standing, while also raising the risk of falls and fractures [3–5]. In children spasticity can be followed by disability, growth problems, and deformed joints [6]. Over 12 million people worldwide suffer with spasticity, including over 80% of those who have multiple sclerosis and cerebral palsy [2, 6]. Spasticity is one of the most prevalent, potentially disabling, and annoying symptoms of spinal cord injury (SCL). Approximately 70% of people with SCL are spastic one year after injury, and roughly half of them receive antispastic medication [7]. Every year, roughly 110,000 people in England suffer a stroke, with spasticity affecting 19–38% of them (up to 41,800 people) [3]. The range of disability caused by spasticity is minimal to extreme. Additionally, there could be variations in spasticity throughout the day, which tend to be more noticeable in those with cervical SCL. Furthermore, 40% of patients who report spasticity may not respond to movement provocation during a physical examination [7].

Associated with the burden of the disease itself chronic conditions deal with the burden of management and treatment of the disease. This is largely due to the ongoing need for treatments intending to ameliorate symptoms or altering the progression of the disease [8–10]. Treatment burden is a significant clinical concern as it can lead to decreased adherence to prescribed treatments and self-care practices, which is strongly associated with adverse clinical outcomes. These outcomes include increased hospitalizations, higher mortality rates, and reduced health-related quality of life [11–16]. In the management of spasticity, treatments are diverse and often used in combination. These may include physical therapy, exercise, medication, surgery, and psychological therapy. Nevertheless, the use of medication has been described as one of the most common treatment forms, with the volume of medications dispensed to the community steadily rising [17]. Despite the availability if these treatments, ensuring adherence to treatment in these patients remains an important challenge in health care where more intensive research is required. Many patients with chronic conditions often need to take medications over extended periods, causing discomfort i.e., “pill fatigue”, side effects and the inconsistent therapeutic effects caused by pharmacokinetic fluctuations. These factors often contribute to higher relapse rates, increased hospitalization, poorer quality of life and higher levels of residual symptoms [18]. From a healthcare systems perspective, patients with chronic conditions represent about 50% of all general practitioner (GP) appointments, over 70% of all inpatient bed days and 64% of all outpatient appointments [19]. Moreover, the expenditures of handling the consequences of poor medication adherence in UK are exorbitant, estimated to be more than $100 billion per year [19]. The global cost of chronic disease was predicted to reach $47 trillion by 2030 [20]. In this scenario, the development of long-acting drug delivery systems presents a promising alternative for the management of chronic conditions, offering the potential to improve treatment adherence and reduce the overall burden on both patients and healthcare system.

Implantable devices have been widely investigated to improve the treatment of multiple diseases, especially for chronic diseases, which need treatment for long periods (more than one month) [21–24]. Even with low drug loadings, these devices can achieve effective delivery and increase patient compliance by minimising potential side-effects [25], consequently enhancing the quality of life of the patients. In chronic conditions this aspect is extremely important because the disease and the treatment are often lifelong [26]. Implantable devices have been investigated for different sites of administration such as subcutaneous, intravaginal, intranasal, intratumoral, intracranial, and intravesical [23, 27]. Despite all the advantages they can produce pain and discomfort at the insertion site [28]. This is due to the process of inserting the implant inside the body. A widely used local anaesthetic agent can be used to reduce pain [29–31]. In the present work, we developed a combined implant containing tizanidine (TZ), for the treatment of muscular spasticity, and LD to reduce the pain at the insertion site [31]. We look ahead to alleviate the burden of chronic diseases and we foresee ongoing innovation. With an implant device, we encourage the patient better adherence to treatment medication with a simple technology that not only will provide a long drug release of tizanidine, but also a local anaesthesia after implant insertion provided by lidocaine.

## Materials and methods

### Materials

Tizanidine base form (TZB) was purchased from Cangzhou Enke Pharma-Tech Co. Ltd. (Cangzhou, China). Tizanidine Hydrochloride (TZHCL) powder was sourced from Tokyo Chemical Industry (The Oxford Science Park, Oxford, United Kingdom). Lidocaine hydrochloride (LD) powder was provided by Fagron UK Ltd. (Newcastle, UK). Poly(vinylpyrrolidone) (PVP) (Plasdone™ K29-32) and hydroxypropyl-β-cyclodextrin (HPBCD) were kindly donated by Ashland (Kidderminster, UK). PCL CAPA™ 6505 (MW = 50 kDa) and PCL (MW = 550 Da) were obtained from Ingevity (North Charleston, South Carolina, U.S.A). Dichloromethane (DCM), methanol (MeOH), trifluoracetic (TFA) and poly(ethylene)glycol PEG (MW = 3,000 Da) were provided by Sigma-Aldrich (Gillingham, Dorset, UK).

### Drug containing pellet preparation

To find the best formulation, pellets containing TZHCL and different excipients were formulated with a mass ratio of 1:1. The excipients evaluated were PVP, Hydroxypropyl-β HPBCD, and PCL. Each powdered blend was uniformly mixed and compressed using a hydraulic press at 1 tonne pressure for 30 s. The composition of the pellets can be seen in Table 1. Melt-processed pellets containing TZHCL-PCL, TZB-PCL, TZHCL-PCL-PEG, and TZB-PCL-PEG, in a mass ratio of 1:1, were also prepared using the Vacuum Compression Moulding (VCM) technique (MeltPrep^®^, Graz, Austria). To ensure a homogeneous distribution of the drug in the pellet, films containing 50% of the drug and 50% of the polymer were prepared by dissolving the mixture with 4 ml of DCM. The formulation was left for one day in a petri dish to allow the evaporation of the organic solvent, and once the film was dried, a piece of it was introduced into the chamber at 80 °C for 5 min. The chamber was then kept closed for another 5 min to cool down. Table 1 shows the composition of the pellets prepared using VCM.

**Table 1:** Composition of the TZ containing pellets

Pellet name	Preparation method	Drug	Drug content (%)	Polymer	Polymer content (%)	Excipient	Excipient content (%)
TZHCL	Direct compression	TZ base	100	-	-	-	-
TZ-PVP	Direct compression	TZ base	50	PVP	50	-	-
TZ-HPBCD	Direct compression	TZ base	50	-	-	HPBCD	50
TZHCL-PCL	VCM	TZ HCl	50	PCL	50	-	-
TZHCL-PCL-PEG	VCM	TZ HCl	50	PCL	25	PEG	25
TZB-PCL	VCM	TZ base	50	PCL	50	-	-
TZB-PCL-PEG	VCM	TZ base	50	PCL	25	PEG	25

### Membrane Preparation and implant assembly

To obtain PCL tubular membranes 1.2 g of PCL (50 kDa), 1.8 g of PCL (550 Da) (40:60) and 30 mL of DCM were mixed to form a solution that was subsequently casted on a spinning metal rod rotating at 75 rpm. The setup was described in a previous work [32]. In brief, a 3 mm metal rod was attached to an IKA RW 20.n (IKA Works Inc., Wilmington, NC, USA) overhead stirrer, positioned at a 45° angle. The rotating rod was dipped into the PCL solution, with three consecutive dips performed to form PCL tubular membranes, allowing 4 min of drying between each dip. Once dried, the membranes were carefully removed from the metal rod. The rods were then left to dry overnight in a fume hood to eliminate any residual DCM. To prepare the reservoir-type implants, the tubular membranes were cut into sections approximately 1.2 cm in length. Subsequently, a single drug-containing pellet (ca. 50 mg in weight) was inserted into the tubular membrane, and both ends were sealed using a pair of pliers preheated to 80 °C, as previously described [32, 33]. Membranes loaded with LD were prepared using a similar procedure. In this case, PCL (50 kDa) and PCL (550 Da) in a 40:60 ratio were dissolved in 30 ml of DCM. LD was then added to achieve a 50:50 LD/PCL ratio. The solution was cast onto a spinning metal rod as previously described, producing three different LD-loaded membranes. To load LD into the outer section of the tubular membrane (final layer), two coatings were applied using a PCL solution without LD, followed by a final coating with the LD-containing solution. Another membrane type was prepared by incorporating LD into the central section (middle layer). This was achieved by first casting with the PCL solution, then with the PCL/LD solution, and finally with another layer of PCL solution. Lastly, a membrane with two LD layers was created using five polymer layers. The first two castings used the PCL solution, the third contained PCL/LD, the fourth was PCL, and the final layer was PCL/LD. These membranes were then loaded with drug-containing pellets and sealed following the protocol described above.

### Characterisation of pellets, membranes, and implants

Physicochemical characterisation of drug containing pellets was conducted to evaluate if the composition of the blend has been affected during the formulation.

#### Microscopic examination

The prepared pellets and assembled implants were inspected using a Leica E24W digital microscope (Leica, Wetzlar, Germany). Additionally, a tabletop scanning electron microscope (SEM) (Hitachi TM3030, Tokyo, Japan) was used to analyse the morphology of the implants. SEM analyses were performed in low vacuum mode with a voltage of 15 kV and no sample pre-treatment.

#### Microcomputed tomography

µCT analysis was conducted using a SkyScan 1275 (Bruker, Germany), at an operating voltage of 50 kV and current of 20 µA with at a pixel/voxel size of 10 μm. Rotational images were reconstructed into slices using Bruker’s NRecon software. Reconstructed slices were set at a threshold capable of identifying the material before bitwise operations were performed to characterise. All scans were reconstructed using the same reconstruction and threshold settings to ensure an accurate comparison.

#### RAMAN spectroscopy

In this study, drug-loaded pellets were analysed using a TA RM5 Raman Microscope (Edinburgh Instruments, Edinburgh, UK) equipped with a 785 nm laser. The settings included a 300 mm pinhole, a 70 mm slit, and full laser intensity (100%). A 10x objective was used to capture the field of view of a cross-section slice of pellets processed with the VCM equipment. A matrix of 20 × 30 points (40 μm separation between points) was used to acquire a Raman spectrum across the pellet cross-section, with a single spectrum recorded at each point using an acquisition time of 3 s. The background of the spectra obtained at each point was removed using the instrument software (Ramacle, Edinburgh Instruments, Edinburgh, UK). Drug distribution was mapped by plotting the intensity of the drug’s characteristic peak at approximately 1350 cm⁻¹. This setup was used for both TZ base and TZ HCl.

#### Differential scanning calorimetry and thermogravimetric analysis

Differential scanning calorimetry (DSC) was used to analyse the implants, as well as the pure drug and polymer. A Q100 differential scanning calorimeter (TA Instruments, Bellingham, WA) was used for the DSC. Scans were conducted from 25 °C to 400 °C, with a heating rate of 10 °C/min and a nitrogen flow rate of 50 mL/min. Additionally, the materials were characterised using a Q500 thermogravimetric analyser (TGA) (TA Instruments, Bellingham, WA). Samples were heated at a rate of 10 °C/min while the nitrogen flow rate was 40 mL/min, and the temperature range for the samples was from 25 to 500 °C.

#### Attenuated total reflectance fourier transform- infrared spectroscopic analysis (FT-IR)

The infrared spectrum of the pellets, membranes, and raw materials was analysed using a Spectrum Two FT-IR Spectrometer (Perkin Elmer, Waltham, MA) equipped with a MIRacleTM diamond attenuated total reflectance (ATR) accessory (PIKE Technologies, Fitchburg, MA). The range used was from 600 cm⁻¹ to 4000 cm⁻¹, obtaining an average of 32 scans with a resolution of 4 cm⁻¹.

#### Powdered X-ray diffraction

A MiniFlexTM X-ray powder diffractometer (Rigaku Corporation, Tokyo, Japan) equipped with Ni-filtered, Cu Kβ radiation and a voltage of 30 kV was used for the evaluation of the crystallinity of the raw materials, final pellets, and implant devices. Continuous mode scanning was carried out at room temperature at a rate of 2°/min throughout an angular range of 3–60° 2θ (2 thetas) with a sample width of 0.03°. The voltage was 30 kV and the current used was 15 mA.

#### Mercury intrusion porosimetry (MIP)

The porosity of each membrane was also evaluated in terms of mercury intrusion porosimetry (MIP). All tests were carried out on an Autopore IV 9500 instrument (Micromeritics, Norcross, GA, USA). The relationship between applied pressure and pore size is defined by the Washburn equation, which assumes a relationship between the applied pressure and pore diameter using physical properties of a non-wetting material (in this case, mercury which has a contact angle of 141° with the test materials). The applied pressure ranged from 1 to 60 000 psi.

### Drug quantification using high performance liquid chromatography

TZHCL and TZB were quantified using reverse-phase high-performance liquid chromatography (RP-HPLC) (Agilent 1100 series system, Agilent Technologies UK Ltd., Stockport, UK). A Phenomenex^®^ LC Column (5 μm particle size, C18 100 Å, 250 × 4.6 mm) from Macclesfield, England, UK, was used to achieve separation of the drug. The mobile phase consisted of a mixture of water with 0.1% TFA and methanol in a ratio of 60:40. The flow rate used was 0.7 ml/min with an injection volume of 10 µl and a total runtime of 12 min. The detector was an ultraviolet (UV) detector set at a wavelength of 227 nm.

For the quantification of LD, an RP-HPLC was conducted (Agilent Technologies 1200 Infinity Compact LC Series, comprising an Agilent degasser, binary pump, auto standard injector, and detector, Agilent Technologies, Stockport, UK) as described previously [29]. A Phenomenex^®^ LC Column (5 μm particle size, C18 100 Å, 250 × 4.6 mm) from Macclesfield, England, UK, was employed to separate the drug. The analytes were separated at 30 °C using a mobile phase containing 0.1% TFA in water and methanol in a 58:42 ratio. The flow rate was 0.7 ml/min, and the injection volume was 50 µl. The analytes were detected at 227 nm absorbance after 5.7 min for TZ and 9 min for LD.

### Release study

In vitro release studies were performed to evaluate drug release from the implants, with and without the PCL membrane, over 150 days. The implants were placed into glass bottles with varying amounts of PBS (pH 7.4), depending on the formulation, at 37 °C with agitation at 40 rpm in the incubator. Drug solubility was evaluated to ensure that sink conditions were maintained throughout the experiment. As a consequence of this, 300 mL of release medium was used for implants containing TZ base, while 10 mL of release medium was used for samples loaded with TZHCl. On the other hand, the release of directly compressed TZ pellets was tested in 15 mL of release media. Samples of 1 mL were taken at different time points and analysed using the previously described RP-HPLC method. To ensure sink conditions were maintained throughout the experiment, the entire medium was replaced with fresh PBS (pH 7.4) at each time point.

### Statistical analysis

Data were reported as mean ± standard deviation. Where relevant, statistical analysis was performed using a one-way analysis of variance (ANOVA) with Tukey’s HSD post-hoc test. Each analysis included a minimum of three replicates. A significance level of *p* < 0.05 was used to determine statistical significance and reject the null hypothesis.

## Results and discussions

### Preparation of membrane

The implants developed in this work consisted of a pellet and a tubular rate controlling membrane formulated with biodegradable polymers. These membranes are prepared using a tubular shape and contained three types of PCLs as described previously [34, 35]. These membranes presented a porous structure that controls drug permeation. In the present powork these membranes were prepared by casting a polymeric solution on the surface of a rotating rod. The resulting membranes had a cylindrical shape with a thickness of approximately 183 ± 6 μm. The thickness of the membranes falls within the range (100–200 μm) reported previously for similar biodegradable rate controlling membranes prepared for implantable devices [36–39].

### Preparation of pellets

**Fig. 1:**
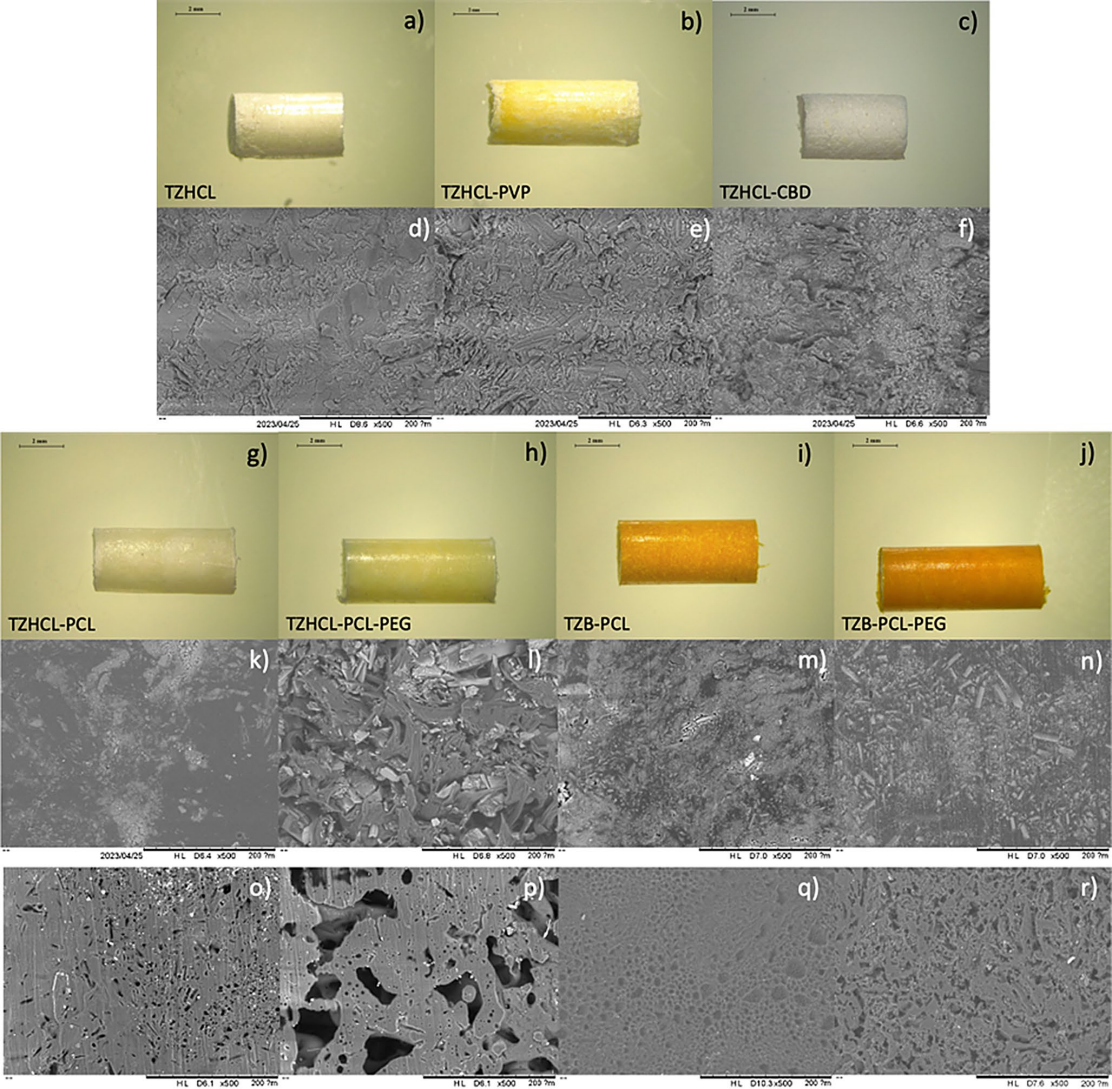
Images of pellets using the Leica E24W microscope at x8 magnification of (**a**) TZHCL only, (**b**) TZHCL-PVP, (**c**) TZHCL-CBD, (**g**) TZHCL-PCL (**h**) TZHCL-PCL-PEG, (**i**) TZB-PCL, and (**j**) TZB-PCL-PEG. Scale bar: 2 mm. SEM images of (**d**) TZ only, (**e**) TZHCL-PVP, (**f**) TZHCL-CBD before release; TZHCL-PCL (**k**) before and (**o**) after release, TZHCL-PCL-PEG (**l**) before and (**p**) after release, TZB-PCL (**m**) before and (**q**) after release, and TZB-PCL-PEG (**n**) before and (**r**) after release at X500 magnification with a scale of 200 mm

Pellets containing TZ and different excipients (PVP, HPBCD, and PCL) were developed by direct compression. The resulting mini tablets have a cylindrical shape designed to be introduced inside the tubular membrane. PCL was selected as a biocompatible, biodegradable polymer capable of controlling drug release over extended periods [40, 41]. Additionally, it is more cost-effective than alternatives such as poly(lactic-co-glycolic acid), helping to keep the overall device cost low [42]. In the case of PCL, the manufacture of the pellets was more challenging. Even though the biodegradable nature of PCL makes it an ideal polymer to prepare implantable devices, this polymer is not suitable for direct compression. Directly compressed PCL pellets did not show good cohesion. It was challenging to remove them from the die and handling them without breaking them. This can be due to PCL properties or to the material particle size that was larger than the one of PVP and HPBCD. Therefore, an alternative manufacturing method for PCL-based pellets was used. Pellets with drug and polymer (PCL or PCL-PEG) in equal mass proportions (50%) were prepared using a melt-processing technique. To ensure a homogeneous distribution of the drug in the implant, a method involving the preparation of drug-polymer films was employed. Subsequently, a portion of the resulting film was melt-processed in the VCM equipment to prepare the resulting drug loaded pellets. The resulting implants took the form of cylindrical rods with dimensions of approximately 2.9 mm in diameter and 15 mm in length (Fig. 1). This size was selected to evaluate implant prototypes. The shape and size of an implant device can significantly impact its effectiveness, safety, and patient comfort [43]. It is important for the delivery method of the device. If the overall size exceeds the capacity for insertion via tools like a trocart, surgical incision becomes necessary. Considering that these pellets are designed to be implanted subcutaneously, they present attributes like a compact size and cylindrical shape to fit in the body with a minimally invasive injection. The implants prepared present similar diameter than commercially available implants such as Nexplanon and shorter length [44]. However, larger implants can be prepared as cylindrical implants of up to 4 cm in length and up to 4 mm in diameter have been described [44]. Indeed, more than one of these pellets can be loaded inside the PCL membranes to provide higher dosages if needed. This could allow the development of implants with different dosage adapted to patient’s needs.

It is important to note that solvent use was included to ensure proper mixing of the drug and polymer. While VCM replicates the melt-processing of hot melt extrusion, it lacks equivalent mixing efficiency. Therefore, solvent casting provides a suitable alternative, as previously reported [45]. DCM has been used in pharmaceutical manufacturing, such as in tablet coatings. However, the FDA advises controlling DCM levels to minimise toxicity risks. Our analysis of similar implants confirmed that residual DCM content remained below the FDA’s recommended limit of 50 ppm [46]. Additionally, cytotoxicity assessments of implants and membranes produced with DCM demonstrated no cytotoxic effects [32, 46, 47]. This can be applied to the membranes described in the "Preparation of membrane" section.

### Effect of excipient on the formulation of TZ hydrochloride and TZ base

#### Microscopy examination (Microscopy, SEM, RAMAN)

The morphology of all the formulations was examined under the microscope. Pellets developed by direct compression presented a rugged surface, related to the powder’s mixture, whereas pellets formulated by VCM had a smooth surface without the presence of bubbles, and the drug was well dispersed throughout the formulation (Fig. 1). The pellets containing TZ in its base form appeared orange due to the presence of the drug. To examine the morphology of the pellets in more detail, SEM was utilised (Fig. 1). The images depicted the presence of TZ and polymer in the all the pellets, with further dispersion evident within their structure, as discernible clusters of drug crystals were not apparent. Interestingly, TZB-based implants appear to have a smoother surface than TZHCl-based implants. This could be because, during the mixing process, both the polymer and the drug were dissolved in an organic solvent to cast films. TZB has higher hydrophobicity, allowing for better mixing and integration with the hydrophobic polymer matrix. Despite this, direct compression and VCM seem to be an appropriate method to prepare drug containing pellets for the development of implantable devices.

One of the most common ways to develop pellets is by extrusion-spheronization [48]. However, VCM and direct compression has been described previously as valuable ways of preparing drug loaded pellets [29, 49–51].

**Fig. 2:**
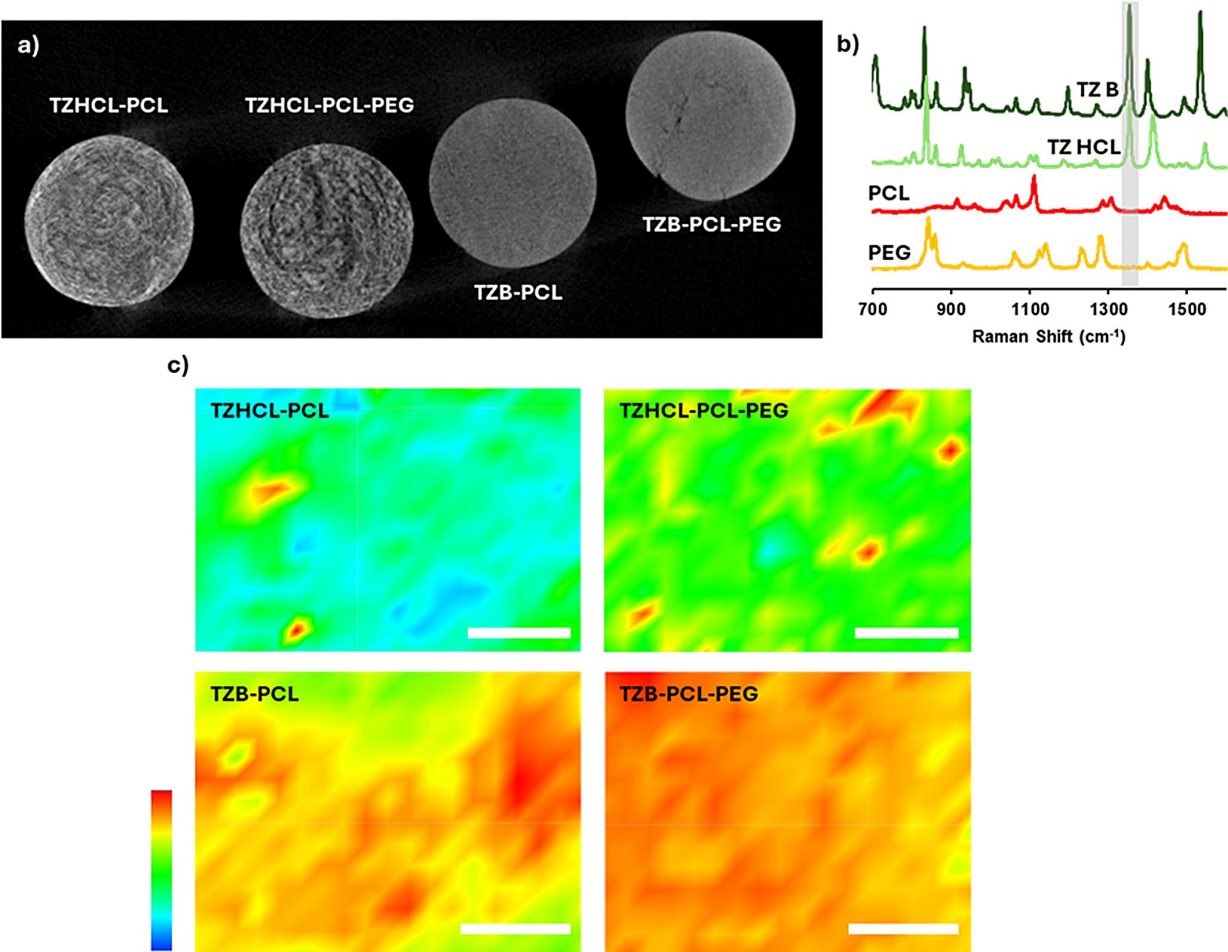
µCT images showing the cross-section of a pellet prepared using VCM (**a**). Raman spectra of the pure compounds used in the preparation of TZ-containing pellets (**b**), and Raman mapping of the cross-section of pellets prepared using VCM (**c**). The white bar in the Raman spectroscopy images represents 200 μm. The colour scale indicates peak intensity for the drug peak at approximately 1350 cm⁻¹, with the minimum representing 0 intensity and the maximum values as follows: 8422 for TZHCl-PCL, 2626 for TZHCl-PCL-PEG, 6560 for TZB-PCL, and 5455 for TZB-PCL-PEG

Moreover, those pellets made by VCM were examinate using MicroCT (Fig. 2a), to verify the lack of holes or bubbles inside the tablets. All the formulations presented a solid core with the drug and polymers well distributed in the matrix, as it was demonstrated previously. Interestingly, the appearance of the pellet cross-sections shows similar results to those obtained in the SEM analysis. TZB-based implants appear smoother than the surface of TZHCl-based implants. This has been attributed to a better mixture between hydrophobic TZB and PCL compared to TZHCl. Additionally, this finding was supported by Raman spectroscopy analysis of the implant cross-sections (Fig. 2b–c). To assess drug distribution within each pellet, we mapped the intensity of the TZ peak at ca. 1350 cm⁻¹, as neither PCL nor PEG exhibited peaks at this specific Raman shift (Fig. 2b). The results suggest that the drug is distributed across the surface of the implant but in TZHCL-based implants there are obvious drug spots (Fig. 2c). This high drug concentration spots are consistent with the roughness previously reported for this type of implants. This phenomenon is more obvious for TZHCL-PCL than for TZHCL-PCL-PEG. The presence of PEG in the later contributes to drug solubilisation within the polymer matrix. Despite the presence of high drug concentration domains, the rest of the surface of the implant contains drug. This behaviour has been observed before for other types of implants that contain drug crystals dispersed within a polymer matrix [52, 53]. Therefore this is not considered a problem in the formulation but this phenomenon might influence drug release kinetics.

#### Thermal analysis: differential scanning calorimetry and thermal gravimetric analysis

**Fig. 3:**
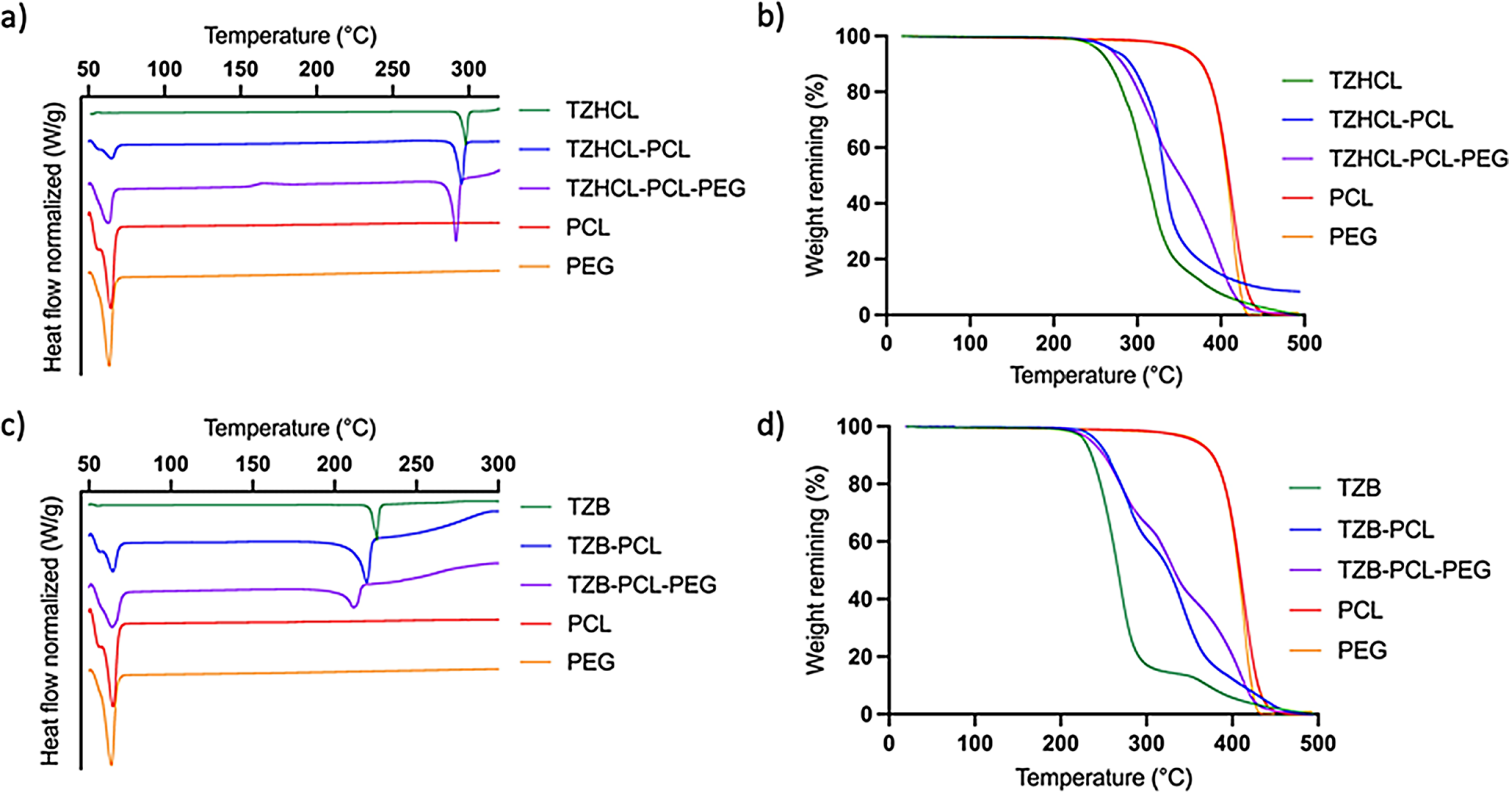
DSC traces for (**a**) pellets containing TZHCL and (**c**) pellets containing TZB. TGA curves for (**b**) pellets containing TZHCL and (**d**) pellets containing TZB

The thermal behaviour of the drug in its isolated form and mixing with polymer, as well as the raw powders of the polymers, were analysed using DSC. Thermograms (Fig. 3) for samples containing PCL and PCL-PEG combinations displayed a distinct endothermic peak at approximately 55 °C, corresponding to the melting points of both PCL and PEG [47, 54, 55]. Notably, when PCL and PEG were combined, the peak became slightly broader, suggesting interactions between the two polymers. These findings align with previous studies on PCL-PEG blends prepared via hot melt extrusion, where PEG was reported to act as a plasticiser for PCL [55]. On the other hand, thermograms, revealed a distinct endotherm peak at 296 °C, corresponding to the fusion of TZHCL, whereas the formulations developed exhibited a noteworthy shift in peak temperature towards lower values (295 °C for TZHCL-PCL and 291 °C for TZHCL-PCL-PEG). This observed difference in the temperatures of fusion between the pure drug and the formulation could be attributed to an interaction between TZHCL and the polymer matrix. On the other hand, the thermogram showed an endothermic peak of TZB at 225 °C, corresponding to the fusion of the drug. The behaviour of the drug base is slightly different as the change in the DSC melting peak is more dramatic. When TZB was combined with polymer (PCL, PEG) the melting peak showed a shift to lower temperatures (219 °C for TZB-PCL and 213 °C for TZB-PCL-PEG) and present a broader shape indicating a reduction in drug crystallinity. Moreover, the effect is more obvious when PEG was incorporated into the mixture. This is consistent with a more even drug distribution obtained in the Raman analysis (Fig. 2). Also, this behaviour was previously reported for PCL/PEG based implants containing olanzapine [56]. Interestingly, drug melting peaks are sharper and more intense for TZHCL-based pellets indicating a higher degree of crystallinity. These results are consistent with the findings reported previously suggesting that the hydrophilic drug salt present domains of high crystallinity within the polymer matrix.

Alternatively, TGA analysis (Fig. 3) shows that the degradation of drug loaded pellets starts at higher temperatures than pure drug confirming the results obtained in DSC. The observed thermal behaviour conforms to the typical patterns exhibited by eutectic mixtures, where interactions such as adhesion forces are prevalent within the system, as documented in previous works [57–60]. These interactions can contribute to reduce drug crystallinity as evidenced by the drug melting point shift to lower temperatures. Equivalent behaviour was reported for other drugs such as risperidone or olanzapine when combined with PCL or PCL/PEG mixtures respectively [34, 61]. Polymer/drug interactions and changes in crystallinity can be evaluated using XRD and FTIR spectroscopy.

#### Attenuated total reflectance fourier transform-infrared spectroscopy analysis (FT-IR)

**Fig. 4:**
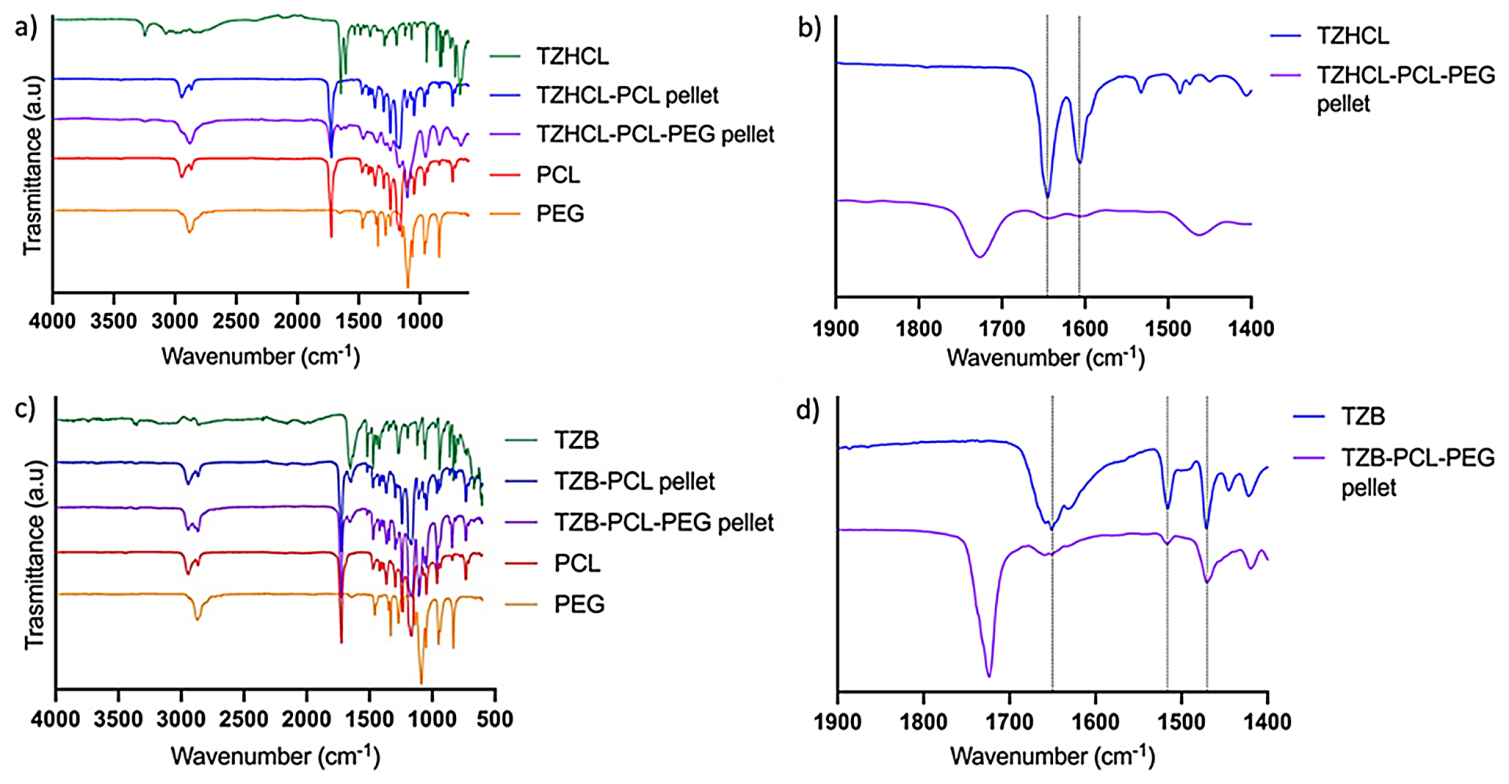
FT-IR spectra of drugs, polymers, and formulation pellets containing TZHCL (**a**-**b**), and TZB (**c**-**d**)

To understand the physicochemical properties and functionality of all formulations, FTIR studies were conducted. Spectra of drug powder, polymers, and mixtures of these components are shown in Fig. 4. Characteristic peaks of TZHCL were observed at 672 cm^− 1^ related to aromatic bending, 945 cm^− 1^ corresponding to aromatic C-Cl stretching, 1645 cm^− 1^ and 1606 cm^− 1^ corresponding to C = C bonds, and 3246 cm^− 1^ related to a secondary amine group [62, 63]. These peaks were evident not only in the spectra of the drug alone but also in the spectra of the pellets, demonstrating the effective integration of the drug into the matrix of the formulated implants. Characteristic peaks of PCL, such as the peak at 1723 cm^− 1^ corresponding to the C = O bond, and 1240 cm^− 1^ correlated to C-O-C stretching [64, 65], as well as PEG indicated by the peak at 1099 cm^− 1^ corresponding to C–O–C stretching [66, 67], were also present in the implants.

The obtained formulations containing PCL only did not show shifts in the absorption bands characteristic of the drug and the polymers, nor did new bands emerge. However, in the TZHCL-PCL pellet, the high intensity in the absorption bands corresponding to the polymer in the mixture appeared to mask some characteristic bands of the drug. Upon detailed analysis of these respective peaks, it was observed that there were no new absorption bands or shifts in the characteristic peaks of the drug, confirming previous observations. Formulations containing a PCL-PEG combination showed a peak shift at 1604 cm⁻¹, suggesting non-covalent interactions between the drug and the polymer matrix. Additionally, a slight shift in the TZHCl amine peak at around 3200–3400 cm⁻¹ was observed in PCL-PEG implants. This shift has been associated with hydrogen bonding between the drug’s amine groups and carbonyl groups [68]. Given that this effect is more pronounced in PEG-containing pellets, we can hypothesise that the hydrogen bonds form between the amine groups of TZHCl and the oxygen groups in the PEG chains. This finding is consistent with the melting point shift presented in the DSC analysis. These results are consistent with previously reported for olanzapine implants prepared using PCL and PCL-PEG combinations [46].

Characteristic peaks of TZB were observed at 1651 cm^− 1^ and 1516 cm^− 1^ corresponding to a C = C bond, and 3355 cm^− 1^ correlated to NH stretching [62, 69]. These peaks are more clearly visible compared to those in TZHCL, in both the pure drug and in formulations with polymers, indicating good integration between the components. Similarly, characteristic peaks of the polymers mentioned before were present in the spectrum. Additionally, TZB-PCL-PEG pellet present a faintly shift of the peak at 1644 cm^− 1^. As it was mentioned before this suggests a non-covalent chemical interaction between the drug and the polymer.

#### Power X-Ray diffraction

**Fig. 5:**
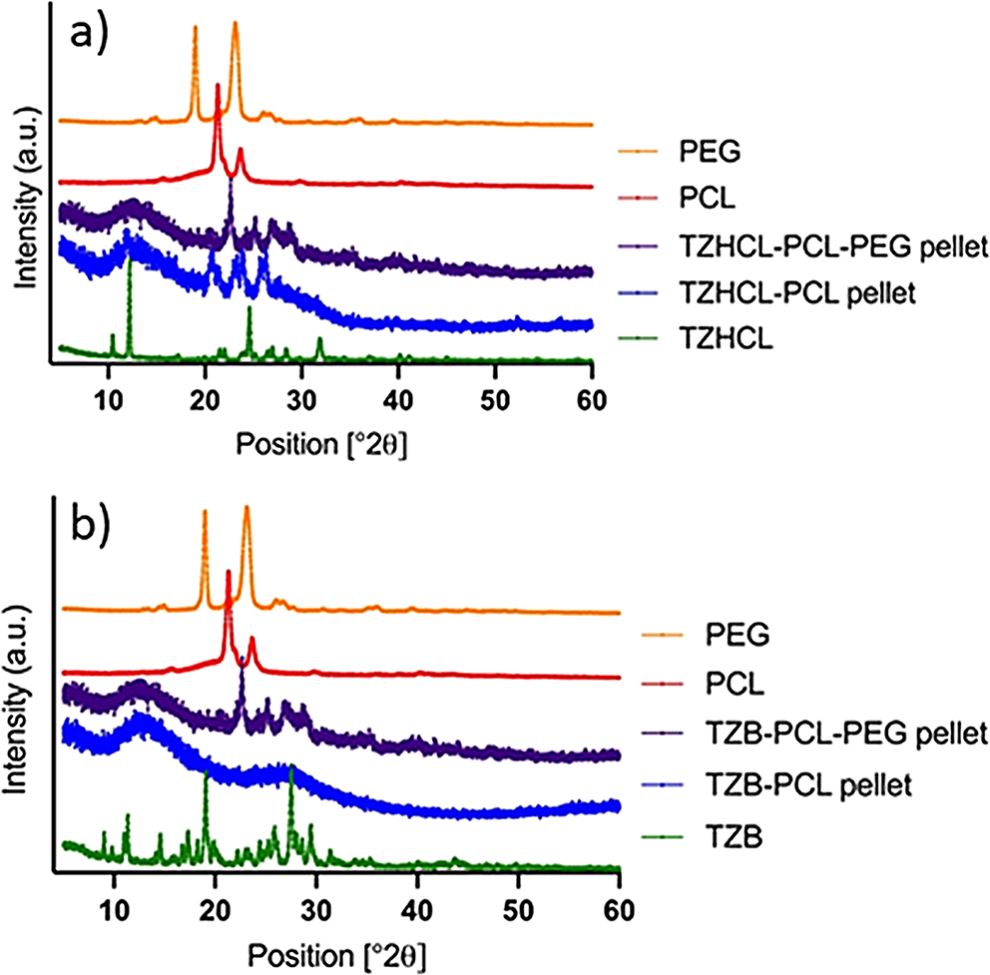
X-ray spectra of drug, polymers, and formulations, containing TZHCL (**a**), and TZB (**b**)

XRD patterns of TZHCL and TZB pellets and the compounds alone are shown in Fig. 5. As we can observed in the figure the x-ray diffraction pattern of the pure drug and the excipients used in the formulation, exhibited reflection characteristic of crystalline systems. Some of the characteristic’s peaks of TZHCL could be seen at 11, 12, 25 and 32 degrees approximately (Fig. 5a). While for the two polymers used, PCL and PEG the characteristic peaks in their diffractogram showed values ​​at 21 and 24 degrees, 19 and 23 degrees respectively. The existence of those peaks in the pellet formulations means that the drug has been well incorporated. The resulting pellets, however, exhibited a notable reduction in the intensity of the pure compounds’ reflection plans along with the loss of other distinct peaks. In the spectrum of TZHCL pellet containing PEG it was possible to observe a partial amorphization with some characteristic peaks belonging to the PEG. On the other hand, the sample without PEG, TZHCL-PCL pellet, present a marked decrease in the intensity of the reflection plans, and disappearance of some characteristic plans for both, the polymer, and the drug, was observed, indicating a partial amorphization of the drug in the system. On the other hand, also pellets containing TZB and the compounds alone exhibited characteristic peaks demonstrating their crystallinity (Fig. 5b). Peaks observed at 9, 11, 19, 28, and 29 degrees corresponded to TZB, while peaks at 21 and 24 degrees were related to PCL, and peaks at 19 and 23 degrees to PEG. The presence of these peaks in the prepared pellets indicates that TZB was thoroughly incorporated into the matrix system. However, there was a noticeable decrease in the intensity of the peaks in the pellet samples, and some peaks were not observable, this could suggest a partial amorphization of the formulation. These results align with the DSC spectrum of the drug demonstrating that the drug still crystalline.

#### In vitro release study of tizanidine hydrochloride

**Fig. 6:**
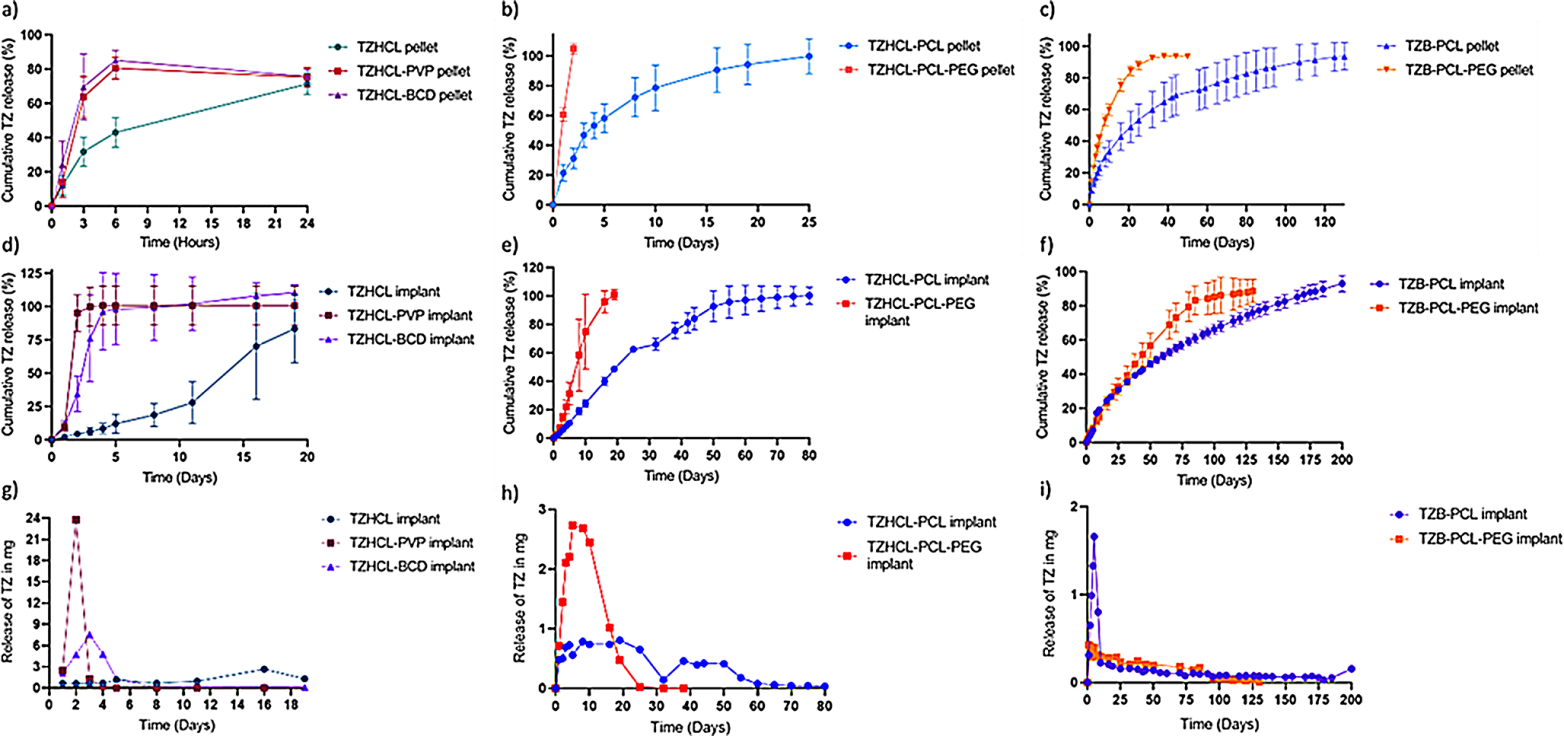
Cumulative release of TZHCL pellets (**a**-**b**), TZB pellets (**c**), TZHCL implants (**d**-**e**), TZB implants presented in percentage of TZ released (**f**), and mg released per day from the TZHCL and TZB implants (**g**-**i**). All results shown as means +/- S.D., *n* = 4

The release profiles of the pellets and implants containing TZHCL in combination with different compounds are presented in Fig. 6. Pellets developed by compression, pellets formulated by VCM, and both types of pellets with a PCL membrane were tested. All formulations evaluated were able to release more than 70% of the drug loaded in the implant device during the experiment period. Compressed tablets without the membrane were unable to maintain drug release for more than 1 day, unlike tablets produced by VCM. Pellets of TZHCL-PCL and TZHCL-PCL-PEG released the drug over 25 and 3 days respectively. Interestingly, pellets containing PVP and HPBCD presented faster releases than TZHCL pellets. HPBCD forms inclusion complexes with a wide variety of compounds while PVP has been extensively used to form polymer/drug complexes [70, 71]. Accordingly, these results are not surprising as these compounds will contribute to increase TZHCL dissolution rate. The direct compression method is widely used for manufacturing oral tablets [72, 73]. Therefore, it is a low cost and easy to scale manufacturing method. It has been reported for the manufacturing of subcutaneous implants for long-acting delivery of HIV drugs [74]. Our primary goal was to load the system with significant amounts of the drug to create a long-lasting implant. Unlike oral tablets, the need for excipients is less critical for this administration route, which made obtaining compressed pellets with short release challenging. On the other hand, VCM pellets are manufactured differently. In this case, mini tablets can be easily developed with the desired shape and homogeneity. The components are exposed to high temperatures until they melt together and create a matrix. The drug dispersed in a polymeric matrix slows down release, as the polymer needs to degrade to expose and release the drug. Although the VCM-pellet containing PCL showed the longest release, its duration is still not promising for a long-acting system device. Hence, a tubular membrane was included as part of the final implant device.

Implants comprising PVP, HPBCD, and the drug alone were inserted into the PCL tubular membrane, and it was observed that the release duration improved compared to pellets alone to 5, 11, and 25 days, respectively. In the case of implants with VCM tablets, the release duration also improved, reaching 80 days for the TZHCL-PCL implant and 19 days for the TZHCL-PCL-PEG implant. Clearly, the tubular membrane helps to control the release of TZ. Similar systems have been reported in the literature [34], but loaded with different drugs and formulation methods.

The pellet formulated with TZB-PCL released the drug by 96 ± 10% over 150 days, whereas the pellet formulated with TZB-PCL-PEG delivered TZ at 94 ± 1% after 50 days. A nearly linear release profile was observed in the first 20 days for the formulation with both polymers, followed by a gradual decrease. Once again, the pellet containing PCL exhibited longer release compared to the formulation containing a mixture of PCL-PEG. These results are consistent with the absence of PEG, a water-soluble polymer, in the implants’ structure. PEG typically creates pores during the release process, aiding in drug delivery.

The surface of the pellets was analysed under SEM after the release study, revealing that the presence of PEG resulted in a more porous polymeric matrix (Fig. 1). This supports the theory that erosion influenced the drug release due to the water-soluble polymer in the matrix. Additionally, the pellets were evaluated in conjunction with the tubular membrane; in this scenario, the TZB-PCL formulation achieved 200 days, releasing 93 ± 5% of the drug, while TZB-PCL-PEG lasted 130 days, releasing 89 ± 7% of TZ. Clearly, the polymeric membrane helps to extend drug release compared to pellets alone. The formulation with PCL showed nearly linear release, maintaining a constant amount of TZ released per day throughout the experiment. Polymeric membranes have been previously employed to develop implant devices, demonstrating their capability to enhance drug delivery.

TZ in both forms, the salt and base, have been evaluated as a model drug for the formulation of an implantable long-acting drug delivery system to treat spasticity related to multiple sclerosis. Current treatments such as oral treatments can lead to systemic side effects like drowsiness, weakness, or gastrointestinal issues [75]. Intrathecal baclofen pumps are effective because they deliver the drug directly into the spinal fluid; however, their placement requires an invasive surgery with ongoing monitoring to avoid complications like infections or pump malfunctions [76]. These subcutaneous implants have distinct attributes over current treatments for spasticity in MS. They are small in size, have the capability to provide a less invasive treatment, and can offer sustained and localised drug delivery for long periods of time. This can lead to more consistent control of spasticity with fewer systemic side effects, as the drug bypasses the gastrointestinal tract, improving the adherence to treatment. Considering the low oral bioavailability of TZ, the formulated implants could be able to release amounts of drug between 0.2 and 3 mg per day which is a significant amount to treat a patient subcutaneously. Furthermore, the implants will not only improve the quality of life of the patient but will also reduce the burden in the health-care system, ameliorating the number of hospitalizations for relapses. Moreover, implants can be development with different drug loadings, shapes, and techniques to offer a more personalised treatment depend on the needs of the patient. Additionally, the maintenance require for subcutaneous implants is less compared to pumps, making them a more convenient option for some patients.

Regarding economic costs of MS, it has been reported that the cost of managing spasticity in MS patients increases with the severity of the condition [77]. Implants could be used early to help control spasticity progression. A UK study estimated that annual spasticity-related costs range from £217 in the early stages to £33,163 in later stages [77]. Early spasticity management has been proven to significantly reduce overall disease management costs [77]. Finally, patient perspectives on implantable devices for MS treatment have been previously evaluated, indicating a preference for this approach over conventional treatments due to the reduced risk of relapse [75]. The study also highlighted that patients would be willing to compromise some treatment efficacy in favour of switching from regular injections to a long-term implant [75].

Interestingly, alternative implants for spasticity treatment exist, such as intrathecal pumps for baclofen delivery [78]. These devices are well tolerated by patients but are active implants incorporating peristaltic pumps, making them expensive. In some cases, the high costs have led to their withdrawal from the market despite their efficacy [78].

Passive subcutaneous implants offer less precise dose control but are more affordable and easier to use. Similarly, Delpor has developed a subcutaneous tizanidine (TZ) implant for maintaining spasticity treatment over 3 to 6 months [79]. These reservoir-type implants are made of titanium with a semipermeable membrane on one side and utilise Delpor’s patented Prozor technology [80]. This system combines the drug with biodegradable polymers that create an acidic environment inside the implant, enhancing drug solubility and enabling zero-order release [80]. In this study, we explore the use of solubility enhancers and pore-forming agents, such as PEG, to modify drug release. The implant developed in this study is fully biodegradable, unlike Delpor’s titanium implants. While degradation will take longer than drug release, meaning implants for chronic use would need to be removed and replaced, this technology could be used for single-use applications, such as post-surgical pain or injection control, without requiring removal. Additionally, PCL and pharmaceutical excipients are more cost-effective than titanium, making the implants described here a more affordable treatment option.

The implants described in this article represent a promising alternative for the treatment of spasticity in MS, especially for those patients who have not success with the conventional treatments.

### Effect of the LD-membrane size in the formulation

#### Examination of the membranes

**Fig. 7:**
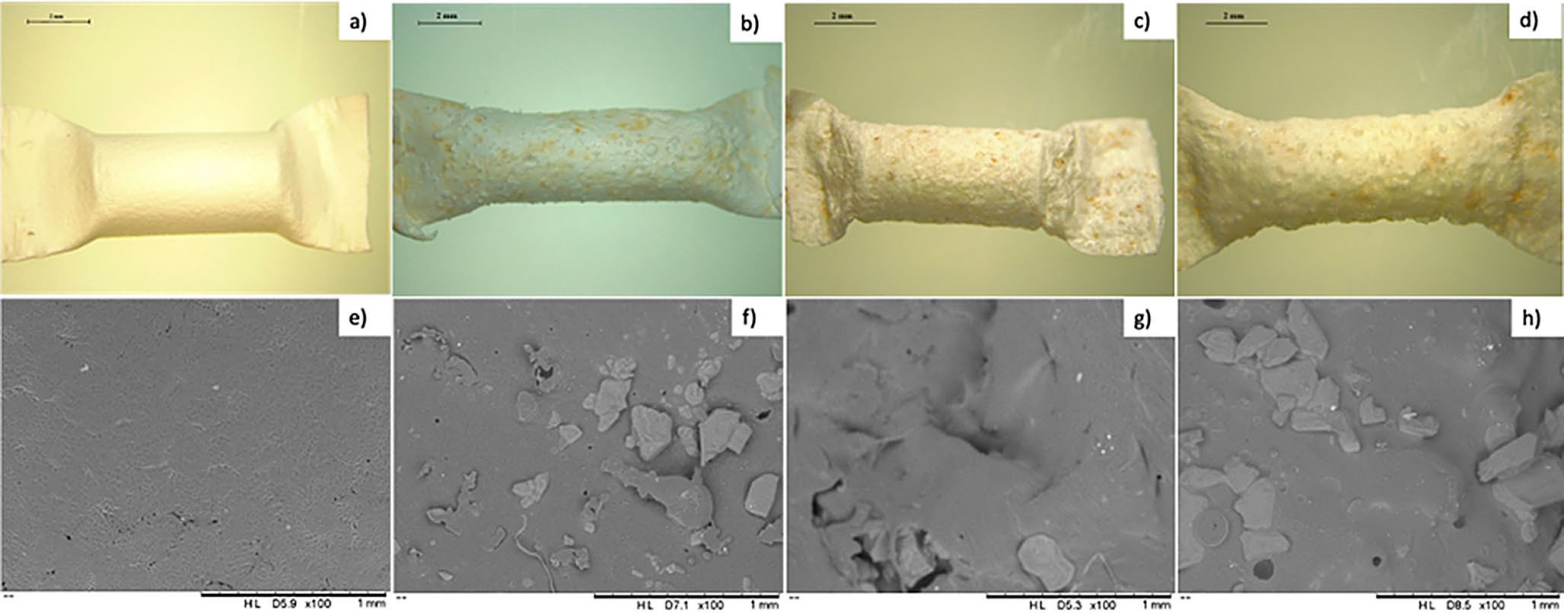
Images using the Leica E24W microscope at x8 magnification of blank tubular membrane (**a**), final implant with a tubular membrane of 3 layers (last layer with LD) (**b**), final implant with a tubular membrane of 3 layers (middle layer with LD) (**c**) and final implant with a tubular membrane containing 2 LD layers (**d**). Scale bar: 2 mm. SEM images at 100 magnifications of blank tubular membrane (**e**), final implant with a tubular membrane of 3 layers (last layer with LD) (**f**), final implant with a tubular membrane of 3 layers (middle layer with LD) (**g**), and final implant with a tubular membrane containing 2 LD layers (**h**). Scale bar: 1 mm

**Fig. 8:**
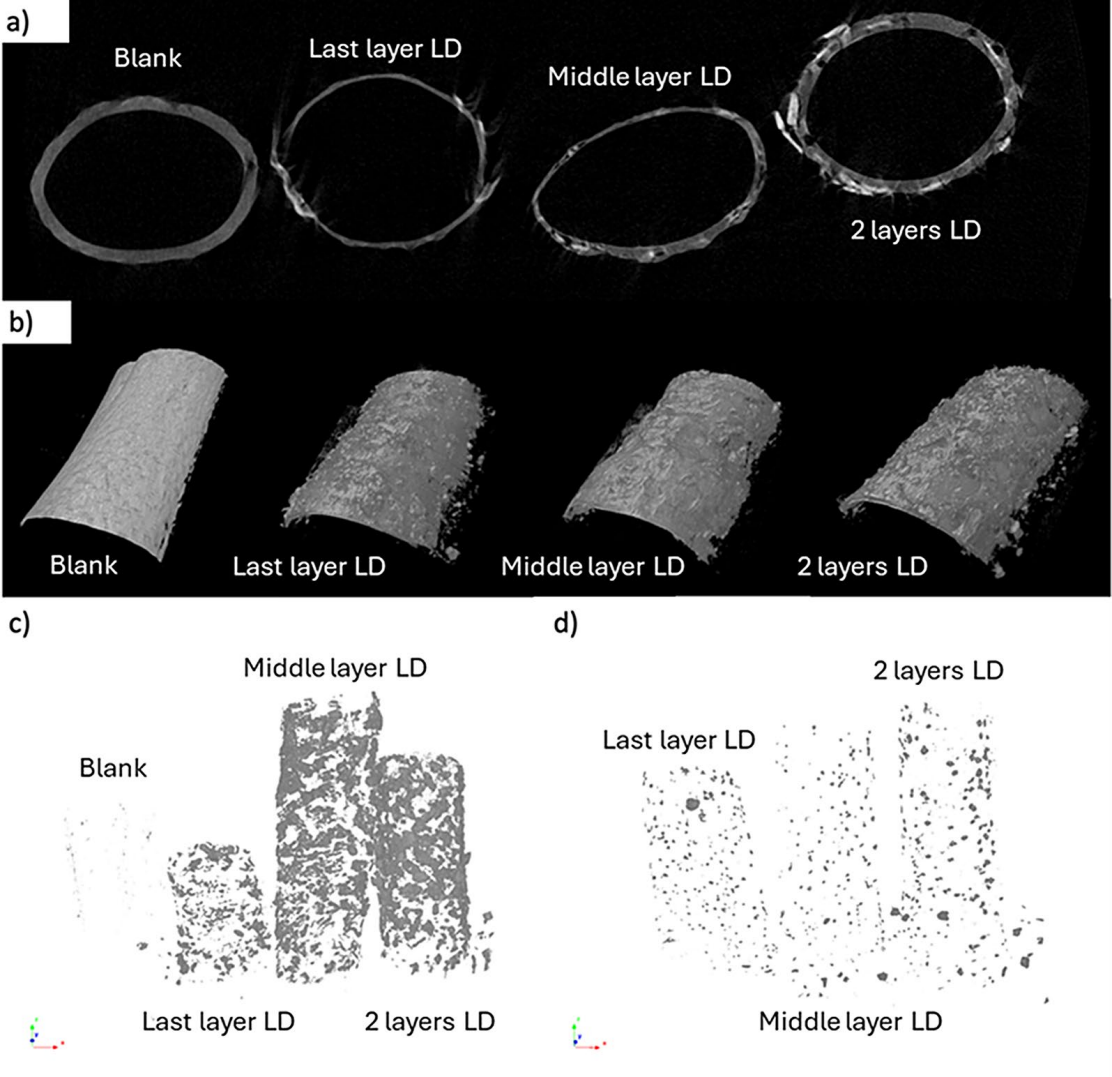
MicroCT of the blank, last layer of LD, middle layer of LD, and 2 layer of LD membrane (**a**). OCT of the surfaces of the membranes with LD (**b**). MicroCT images of membranes containing LD before release study (**c**), and membranes containing LD after release study (**d**) highlighting high density regions

Subcutaneous implants offer multiple obvious advantages, the first of which is their long duration of action [35]. They are also user-independent, indicating that once implanted, the device performs automatically and does not require active participation or input from the patient to administrate the drug [27, 81, 82]. However, they present disadvantages of which the most prevalent is their complex insertion procedure. In these cases, a healthcare professional is required, as well as a small surgical proceeding. Despite the use of anaesthesia patients may still experience pain or discomfort after the implant insertion. It has been reported than more than 80% of surgery patients experienced severe postoperative pain [83]. Acute pain after surgery typically subsides completely in 1–2 weeks with effective therapy [30]. However, for many people, acute postsurgical pain lasts throughout the normal time for tissue repair and evolves to a “chronic” or persistent pain condition. Subcutaneous implants containing LD on their external membrane, were developed to ameliorate the pain after the implant placement. Three different membranes were manufactured: one of 3 layers, with the last layer containing a mixture of PCL and LD, other of 3 layers with the middle layer containing PCL and LD, and the other containing 2 LD layers, where the first and the third (from outside to inside) have a PCL-LD blend. It was possible to load the polymeric tubes with approximately 1.5 mg, 3.3 mg and 6.4 mg of LD, respectively. The tubular membranes of PCL and the mixture between PCL and LD were examined using different techniques. When LD is added to the layers of the tubes, the texture of the membranes differs from those made solely with polymer, as shown by optical microscopy and OCT techniques (Figs. 7a, b and c and 8b).

Moreover, using MicroCT technology, it was clearly seen that LD was added, and it was feasible to observe the different thicknesses of the membranes given by the number of layers of PCL they contain. Images before and after the release of the membranes were taken (Fig. 8c and d). The grey zone in the figure shows the amount of drug before the release experiment, and it is evident that it is evenly distributed throughout the polymer tube. Meanwhile, after the drug release, the grey dots are smaller and in fewer amounts, indicating the release of the drug. We could conclude that the addition of LD into the tubular membranes was successfully achieved.

#### Mercury intrusion porosimetry (MIP)

**Fig. 9:**
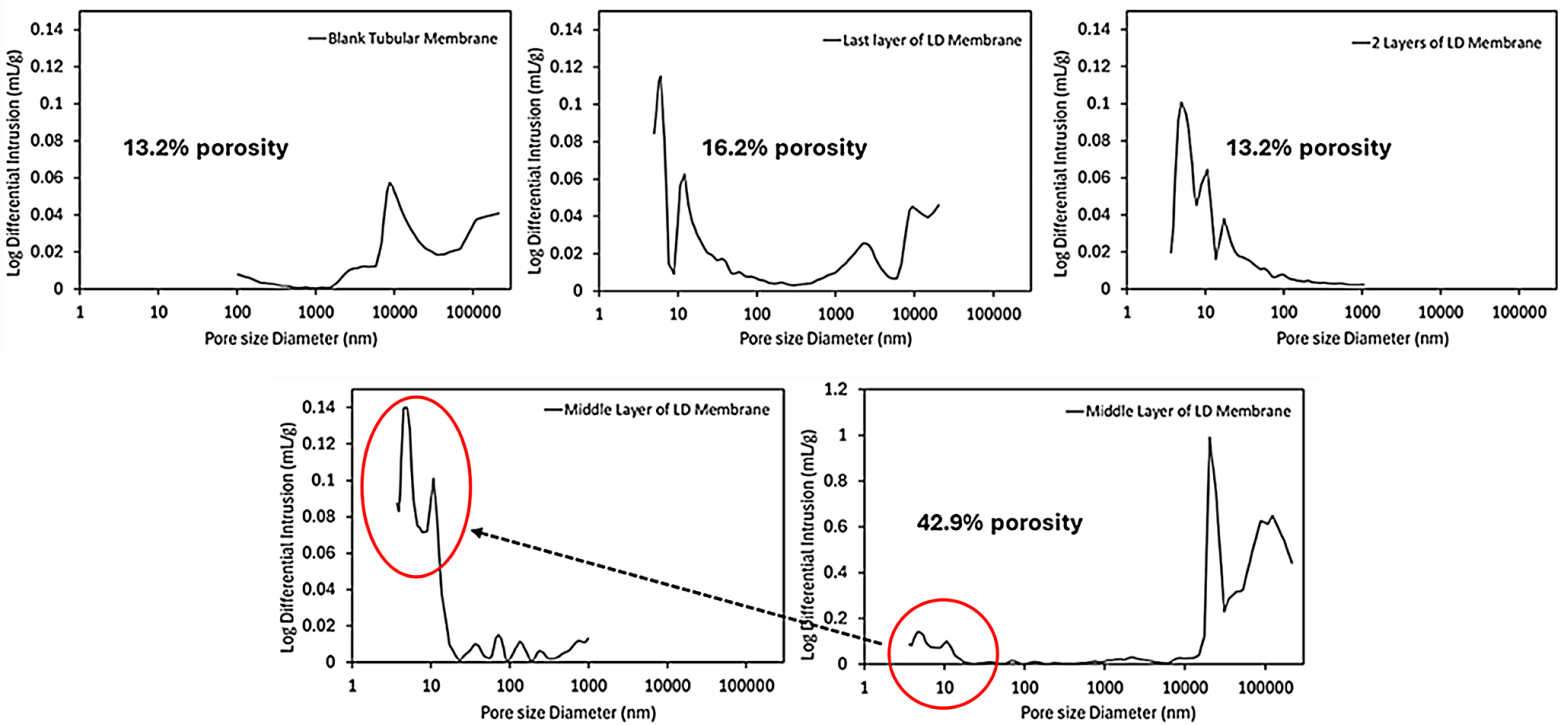
Pore size distribution curves of blank and LD-loaded tubular membranes

The porosity of the membranes was assessed using Mercury Intrusion Porosimetry (MIP) (Fig. 9). The blank tubular membrane exhibited a monomodal pore size distribution, with a peak centred around 10 μm. This pore distribution aligns with previously reported PCL-based membranes, which had the same composition but between one and four additional layers (4 to 7 instead of 3) and showed pores around 1 μm [32]. As expected, the addition of more layers likely decreases the final pore size distribution of the membranes. When LD was incorporated into either the middle or outermost layer, a bimodal pore size distribution was observed in both cases. Specifically, when LD was embedded in the middle layer, two distinct pore size distributions were detected: one between 0.005 and 0.010 μm and another with a significant proportion of pores around 20 μm, extending up to 100 μm. Similarly, when LD was incorporated into the outermost layer, the pore size distribution comprised a lower range between 0.006 and 0.010 μm, along with a higher pore size distribution ranging from approximately 2.5 to 10 μm. Interestingly, the total porosity was higher in the membranes where LD was placed in the middle layer, as the proportion of larger pores (around 20 μm or more) was considerably greater than that of smaller pores (Fig. 9). In contrast, when LD was incorporated into the outermost layer, the distribution was more balanced, leading to a lower overall porosity (Fig. 9). On the other hand, the tubular membrane, composed of five layers—of which two (the first and third, from the outer to the inner surface) contained a PCL-LD blend—exhibited a significantly narrower pore size distribution range compared to the previous cases, with pore sizes ranging only between 0.005 and 0.020 μm. These results align with the porosity percentages obtained from the porosimetry analysis. Thus, it can be inferred that increasing the number of layers from three to five, along with incorporating lidocaine into two layers instead of one, significantly influences both the reduction in pore size distribution and the overall porosity percentage.

#### Thermal analysis: differential scanning calorimetry and thermal gravimetric analysis

**Fig. 10:**
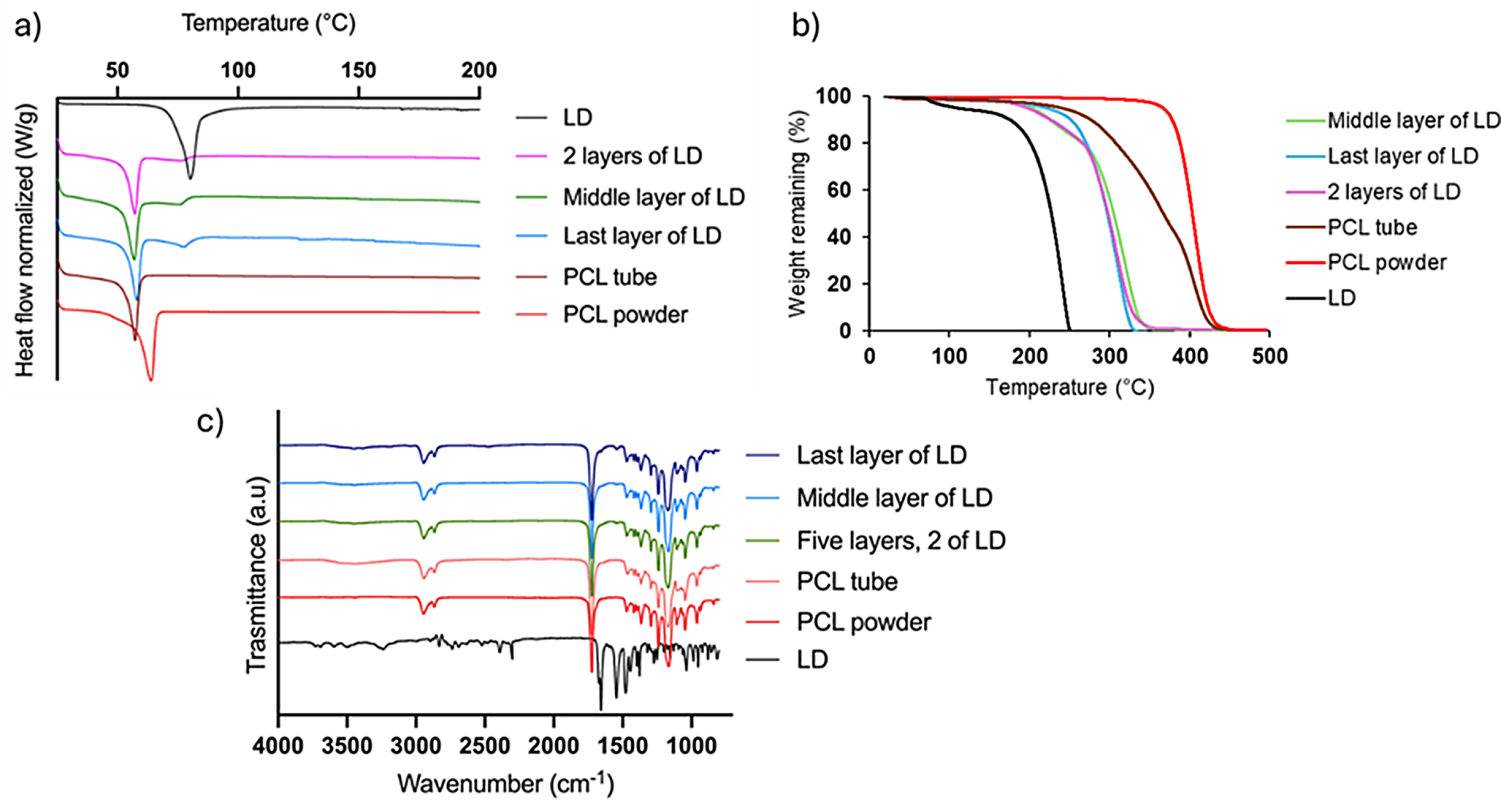
DSC (**a**) and TGA (**b**) curves of tubular membranes and FT-IR of PCL tube with and without LD (**c**)

Figure 10a shows the DSC curves along with their corresponding TGA thermograms of LD, PCL, and the membranes obtained. The characteristic fusion peak of PCL is observed at 65 °C, and the DSC curve of LD shows an endothermic peak at 81 °C, corresponding to its fusion peak [34, 84, 85]. The endothermic peak of the blank tubular membrane was obtained at 58 °C, related to the fusion peak of PCL. This shift to lower temperatures could be attributed to the rearrangement of the polymer molecules into different crystalline phases upon the removal of the total organic solvent (DCM) during membrane formulation. The same behaviour could be observed in the samples containing LD. Moreover, the samples containing LD (2 layers, middle layer, and last layer of LD) showed a fusion endotherm at approximately 79 °C, which would correspond to the peak of the drug. This shift of the pure drug in the membrane samples could be due to a physical type of interaction between PCL and LD, concluding that the crystalline state of both the drug and the polymer used were not altered in the formulation obtained.

Regarding the thermogravimetric analysis of pure LD (Fig. 10b), it showed a mass loss around 100 °C, which could be attributed to the loss of water molecules that could be retained in the pure drug [86]. Following this event, a significant loss of mass was observed at 175 °C, indicating that the drug is thermostable up to 174 °C. PCL powder showed an abrupt loss of mass that began at 360 °C; therefore, it can be concluded that the polymer is thermally stable up to this temperature. Interestingly, PCL-based membranes exhibited lower thermal stability due to the addition of low molecular weight PCL. This behaviour is expected, as changes in the crystallinity of PCL membranes when combining high and low molecular weight polymers have been previously reported [87]. Unsurprisingly, the degradation temperatures of LD-containing membranes were lower than those of PCL tubes without the drug. This effect was more pronounced in tubular membranes containing two LD layers as the double layered LD membrane contained a higher percentage of drug as reported previously.

#### FT-IR spectroscopy

The physicochemical properties of LD and PCL were assessed using FT-IR. Figure 4 displays the infrared spectra of LD powder, PCL powder, and the tubular membranes containing PCL and a mixture of PCL and LD. The formulations of the membranes containing LD demonstrate similar behaviour. The characteristic bands of the polymer were more prominent in the spectra than those of the drug. However, none of the characteristic bands of LD and PCL disappeared or shifted towards longer or shorter wavelengths. Furthermore, none of the tubular membranes showed the appearance of new infrared bands. This can be attributed to the low LD content compared to PCL in the resulting membranes.

#### Release study

**Fig. 11:**
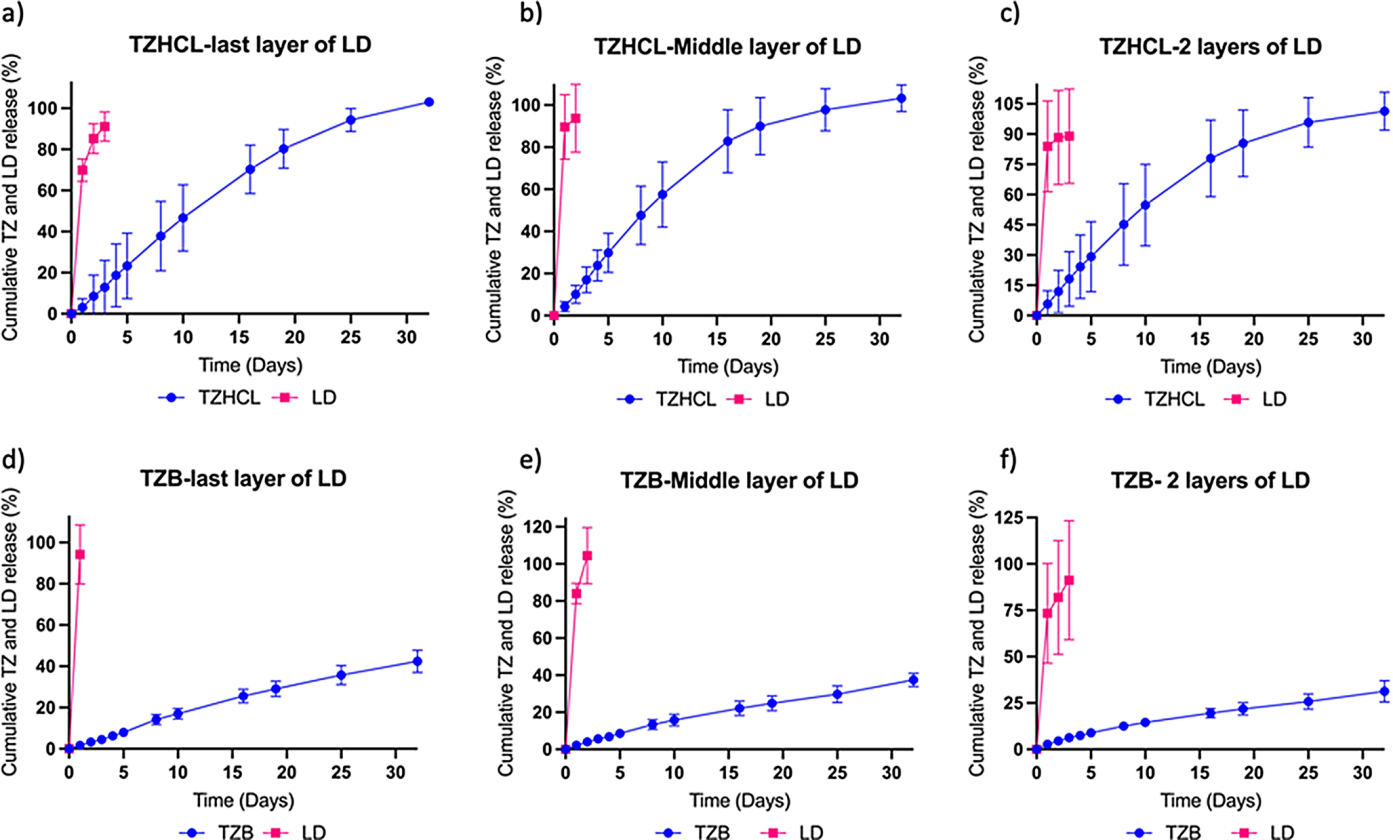
Cumulative release of TZHCL implants (**a**-**c**) and TZB implants with three different LD membranes (**d**-**f**). All results shown as means +/- S.D., *n* = 4

Implants containing TZ in its salt and base forms were tested in an in vitro release study, this time incorporating the three different tubular membranes loaded with LD previously described (Fig. 11). This experiment ran for 32 days to understand the behaviour of the drugs when used together. Formulations carrying a tubular membrane with the last and middle layer made of PCL-LD showed a total duration between 1 and 3 days for LD and the drug released was more than 90% in all the formulations. Conversely, implants made with tubular membranes comprising 2 layers of LD were able to release LD for 3 days, with amounts released being 89 ± 23% and 91 ± 32% for implants encapsulating pellets of TZHCL and TZB, respectively. Considering that LD was added to relieve pain after implant insertion, this duration of drug delivery is enough for local anaesthesia [88–90]. The behaviour of TZ in the release study is consistent with the previous results. The total amount of TZHCL released from the implants was 103 ± 2% (las layer of LD tube), 103 ± 6% (middle layer of LD tube) and 101 ± 9% (2 layers of LD tube) after 32 days. During the same period of time, the implants delivered TZB in amounts of 42 ± 5% (las layer of LD tube), 37 ± 3% (middle layer of LD tube) and 31 ± 6% (2 layers of LD tube). The difference between the formulations is due to the nature of the drug (salt and base). Finally, these constructed implants exhibit the desired features with a small size to use as a long-acting implant by subcutaneous via while also providing a novel advantage in the form of local anaesthesia.

**Fig. 12:**
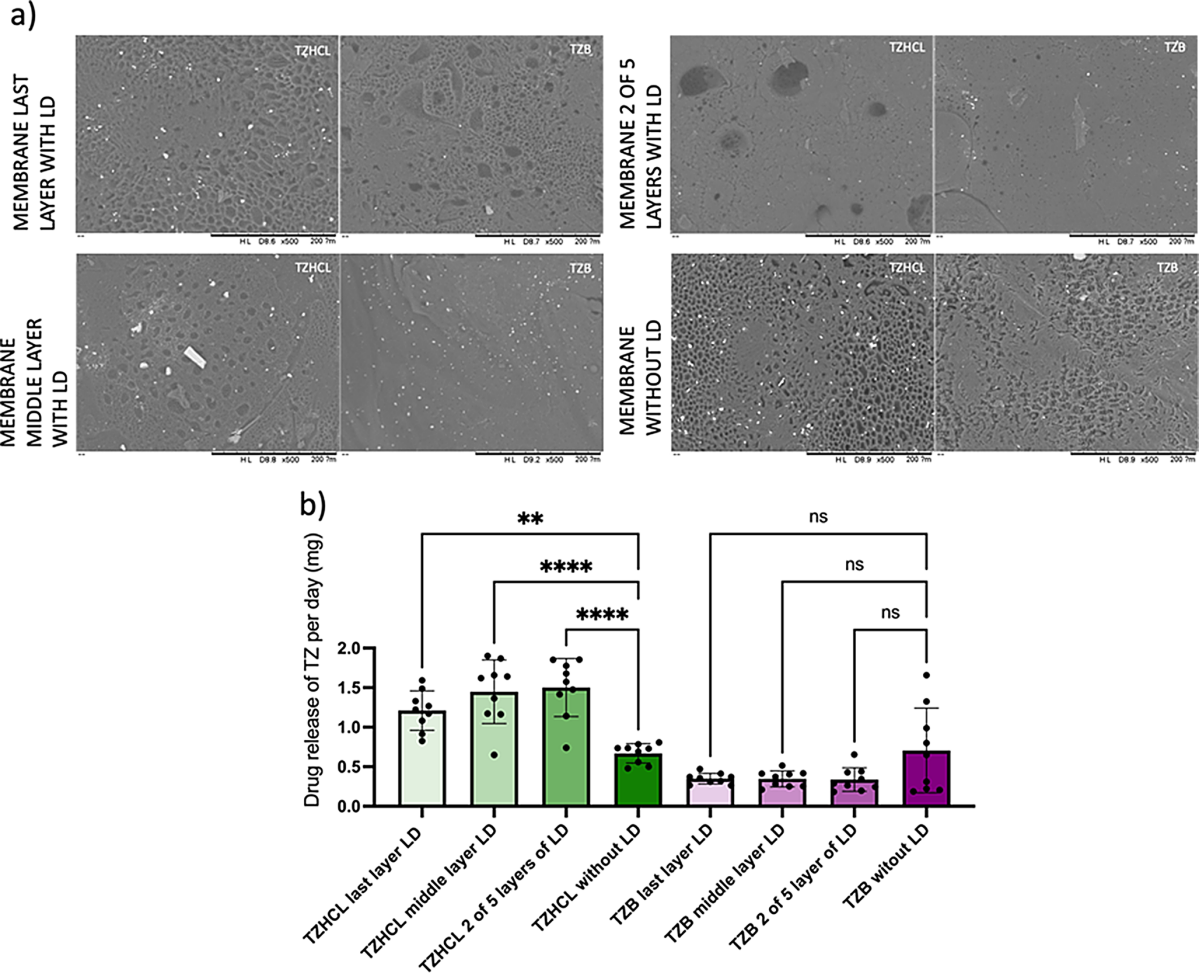
SEM images at x500 magnification of the different membranes containing pellets of TZHCL and TZB after release (**a**). Comparison of mg of TZ released per day between implants with and without LD (**b**)

Comparisons of mg per day of TZ, released from different types of membranes were performed (Fig. 12). Implants comprising TZHCL released more drug in the presence of an LD-coated membrane, whereas those loaded with TZB showed no significant difference between the types of membranes. On the other hand, the behaviour of the release of both forms of TZ (salt and base) in the same type of membrane demonstrate to have significant difference when the membrane has LD on it. However, implants made only with PCL presented no significant difference between TZHCL and TZB pellets.

The development of implants containing lidocaine has been reported in the literature. An example from Liu. D. et al. described the use of a hydrogel implants able to load up to 4 mg of the drug and released LD in a range between 0.48 and 2.4 mg [91]. Whereas these implants were aimed at treating pain after a dental surgery, the implants reported here are designed to treat local pain subcutaneously. This makes both pharmaceutical forms significantly different from one another. Furthermore, another publication presented an implant where LD is part of a polymeric matrix [29]. In this work the authors developed polymeric rods containing 23 mg of the drug, releasing 85% of it after 30 days. Clearly, loading the drug in a matrix versus in a membrane allows for higher drug loadings, when compared to the membranes implant reported here. Crucially, the use of the novel membranes would lead to a more readily release of the drug for shorter and more clinically relevant periods of time. Injectables implants also have been developed [92], leading to a release profile of 5 days, delivering LD up to 85%. In this ork, we presented a new way to deliver LD to treat pain after implant insertion, in which LD can be combined with other drug, like TZ to treat spasticity and at the same time relive pain at the insertion site for 5 days post-operation. Moreover, this drug delivery system device provides an alternative platform to be used to treat chronic conditions.

## Conclusions

This work has demonstrated the possibility of formulating a long-acting drug delivery implant containing two different drugs, one to treat spasticity in MS, and the other to alleviate pain after the implant insertion. The subcutaneous implants consisted of a cylindrical pellet loaded with TZ and covered with a polymeric membrane containing LD. Two different techniques were evaluated to manufacture the implants: direct compression and VCM. The second technique has been the best option for achieving the desired characteristics of the pellets. A biodegradable tubular polymeric membrane, formulated with PCL, was then applied to the implants to act as a rate-controlling barrier. These membranes not only were tested with the pellets inside, but also with the addition of LD in between the layers of the tubes. All formulations underwent physicochemical characterization, demonstrating homogeneity and non-covalent chemical interactions between the compounds. Furthermore, the in vitro release studies demonstrated faster release of TZHCL implants compared to those containing TZB. This is due to the nature of the drug; the salt form is more soluble than the base form. The addition of PEG in the formulations showed an increase in drug delivery since the polymer acts as a co-solvent of the drug, providing faster drug release. All the implants demonstrated the ability to deliver TZ. This suggests a novel implantable formulation that could potentially be an alternative for treating spasticity in MS. These drug delivery device systems have the advantage of not only delivering the drug to treat the disease but also providing local anaesthesia with the same implant device. Therefore, further research like in vivo studies is needed to assess the delivery of TZ and LD from the subcutaneous implant to the body.

## Data Availability

The datasets generated during and/or analysed during the current study are available from the corresponding author on reasonable request.
