# Correction: Preparation, characterisation, and testing of reservoir-based implantable devices loaded with tizanidine and lidocaine

**DOI:** 10.1007/s13346-026-02047-3

**Published:** 2026-02-24

**Authors:** Camila J. Picco, Mihir S. Bhalerao, Octavio E. Fandino, Elizabeth R. Magill, Qonita Kurnia Anjani, Jonathan G. Acheson, Ryan F. Donnelly, Juan Domínguez-Robles, Eneko Larrañeta

**Affiliations:** 1 School of Pharmacy, Queen’s University Belfast, Belfast, UK; 2 School of Engineering, Ulster University, Belfast, UK; 3 Department of Pharmacy and Pharmaceutical Technology, Faculty of Pharmacy, University of Seville, Seville, 41012 Spain

**Correction: Drug Delivery and Translational Research (2025) 15:4708-4728**

10.1007/s13346-025-01855-3

The authors regret that the XRD curve for TZHCL-PCL-PEG (Fig. 5a) was inadvertently duplicated as the TZB-PCL-PEG curve. The corrected Fig. 5a, now including the accurate TZHCL-PCL-PEG XRD curve, is provided below. This correction does not affect the discussion or the conclusions presented in the article.

Additionally, the daily release rates for TZB-PCL implants (Fig. 6i) were not plotted correctly. The corrected Fig. 6i is shown below. The main discrepancy was in the representation of the release rate during the initial days.

These initial release rates were used as a control in Fig. 12b, which has now been updated accordingly. It is important to note that the only change in this figure concerns the release rate for TZB without LD. The corrected results indicate a lower degree of variability in the release rates for TZB-PCL implants. As shown in the revised Fig. 12b, this does not affect the statistical analysis. The corrected data exhibit less variability, and the conclusions of the manuscript remain unchanged.

Overall, these corrections do not impact the description, interpretation, or conclusions of the study.


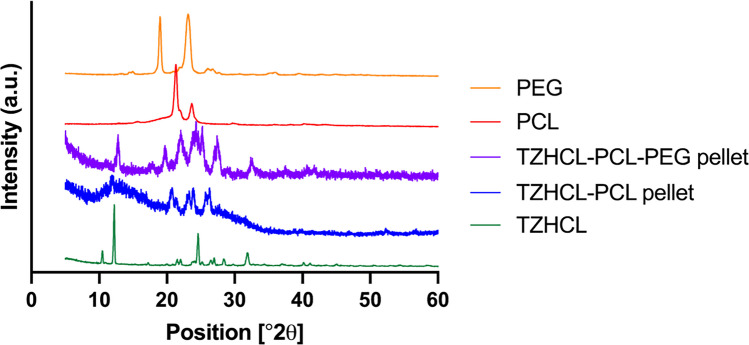


**Fig. 5a.** X-ray spectra of drug, polymers, and formulations, containing TZHCL


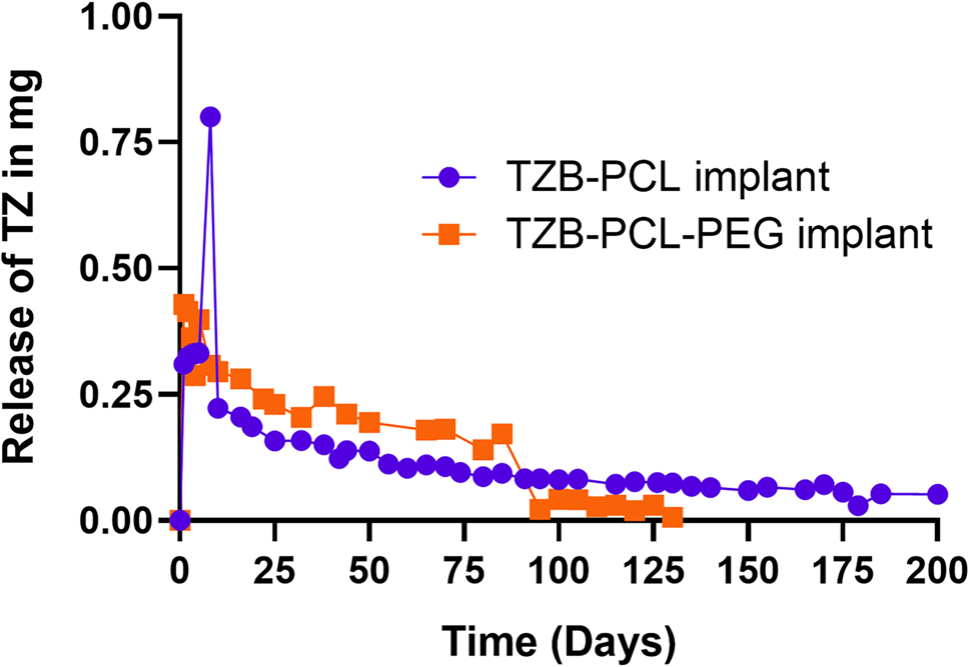


**Fig. 6i.** TZB release rate expressed in mg per day from TZB implants


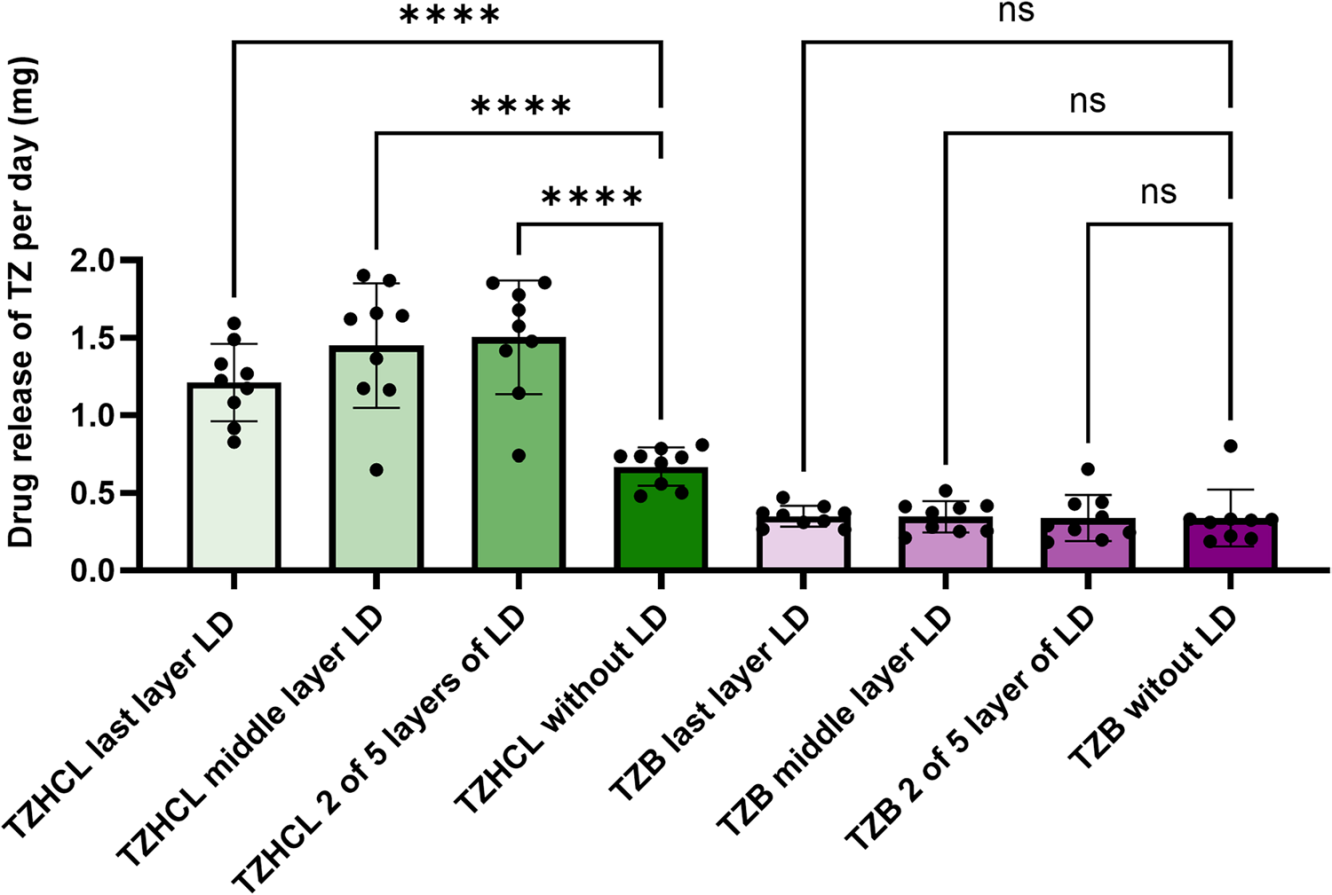


**Fig. 12b.** Comparison of mg of TZ released per day between implants with and without LD

